# Panel interview for internal medicine residency selection: pros and cons

**DOI:** 10.5116/ijme.58a8.0234

**Published:** 2017-02-25

**Authors:** Yasir Illahi, Ghattas Alkhoury, Zubair Khan, William Barnett, Ragheb Assaly

**Affiliations:** 1 Department of Internal Medicine, The University of Toledo, Toledo, OH, USA

**Keywords:** Panel interview, internal medicine, residency selection, USA

## Introduction

Selection of residents is an extremely important process for a residency program as it determines the quality of patient care as well as the academic and research progress of the program. Most studies have focused on the relationship between the national board exam scores, such as the United States Medical Licensing Examination (USMLE) and the subsequent clinical performance of the selected residents. However, a low to moderate correlation has been found between board scores and residency performance.^1^  

Additionally other studies have assessed the residency program directors’ opinions about the relative importance of various selection strategies. They found the top three common selection elements were clerkship grades, reference letters, and the USMLE Step 1 scores.^2^

There is no consensus as to which selection strategy is the most useful in choosing the candidates. Thus, the existing selection process is not ideal and has many shortcomings. For instance, it may not give an accurate representation of the interviewee as a medical resident.^3^ Likewise, there may be a major reliance on one parameter, such as the USMLE scores. This may come at the expense of other parameters, such as the candidate’s communication skills, character, and professionalism.^4^ Also, there is inter- and intra-observer variability in rating the residency candidates. These shortcomings make the selection process subjective and unreliable, which should be avoided when making assessments.^5^

### Adopting a new interview style

During the 2015-2016 interview season, the University of Toledo Internal Medicine Residency Program introduced a panel-style interview. Previously, the residency program relied on one-on-one interviews with various faculty members to evaluate candidates. Changing the interview style allowed an opportunity for multiple interviewers (the panel) to evaluate a residency candidate. Our panel members consisted of the program director/associate program director, a faculty member, and the chief resident. Also, under the one-on-one interview format, it allowed the interviewer greater access to beforehand knowledge of the candidate, which was changed under the panel interview style. Instead, the interviewers were blinded to the objective criteria, such as USMLE scores. After completion of the interview, the panel members individually scored the residency candidate using various subjective elements, such as communication skills, teaching potential, and professionalism. Subsequently, these scores were used by the residency program to calculate the final rankings of all the candidates during the interview season.

### Lessons learned

The panel-style interview approach was designed to emphasize a balanced assessment tool that takes into account both the objective and subjective aspects of the residency candidate. We found that the quality of medical school was the strongest objective factor in the final ranking of the candidates. We question whether this was due to our arbitrary scoring system that might give too much advantage to US programs over international schools. Likewise, evaluators might show some bias toward residency applicants from our medical school. Another strong factor was the subjective component of the scoring system, which came ahead of the USMLE scores. We considered the fact that since panel members were blinded to objective factors, they were less likely to anchor too heavily on USMLE scores and dismiss the other qualities revealed during the interview when it was time to score the candidate. Another pertinent finding was that the program director/associate program director, faculty members, and the chief resident scored the candidates similarly when assessing the subjective elements. This suggests the panel members were consistent in how they viewed the candidate’s professionalism, teaching potential, and communication skills.

## Conclusions

The residency interview process varies across different academic programs around the US and in other countries. Some residency programs may give more weight to the subjectively assessed potentials of the candidates.

Conversely, some residency programs may rely more on the objective parameters, such as USMLE scores, quality of medical school, academic performance, and scholarly activity. In either case, interviewing candidates is a complex process for residency programs and should include a multitude of assessment tools. We believe our panel style interview approach to evaluate internal medicine residency candidates is the first step in the development of both a standardized and balanced assessment tool at our institution.

### Conflicts of Interest

The authors declare that they have no conflict of interest.
